# Characteristics and predictors of stroke mimics in young patients in the norwegian tenecteplase stroke trial (NOR-TEST)

**DOI:** 10.1186/s12883-023-03425-x

**Published:** 2023-11-15

**Authors:** Eskil Jacobsen, Nicola Logallo, Christopher Elnan Kvistad, Lars Thomassen, Titto Idicula

**Affiliations:** 1 Norwegian University of Science and Technology, Trondheim (NTNU), Trondheim, 7034 Norway; 2 Department of Neurology, Centre for Neurovascular Diseases, Haukeland University Hospital, Bergen, 5021 Norway; 3 Department of Clinical Medicine, University of Bergen, Bergen, Norway; 4 Norwegian University of Science and Technology (NTNU), Trondheim, 7034 Norway; 5 Department of Neurology, St Olav University Hospital, Trondheim, Norway

**Keywords:** Stroke, Acute ischemic stroke, Stroke mimics, Mimics, Intravenous thrombolysis, Tenecteplase, Alteplase

## Abstract

**Background:**

Several studies have shown that stroke mimics occur more often among young patients. Our aims were to identify the common mimics in young patients under the age of 60 years who received thrombolysis, to analyze the risk of hemorrhage after treatment with thrombolysis, and to identify risk factors and clinical parameters that might identify mimics in this group.

**Methods:**

Norwegian Tenecteplase Stroke Trial was a phase-3 trial investigating safety and efficacy of tenecteplase vs. alteplase in patients with acute ischemic stroke. Patients diagnosed with either acute cerebral ischemia or transient ischemic attack were categorized as stroke group, and patients with any diagnosis other than ischemic stroke or transient ischemic attack as mimics group. Patients were grouped post-hoc into young (< 60 years) and old (≥ 60 years). Logistic regression analyses were performed with mimics vs. stroke as dependent variable to identify predictors of mimics.

**Results:**

Of the 1091 patients included in the trial, 211 patients (19.3%) were under the age of 60 years. Out of the 1091 patients, 434 (39.8%) were female, median age 77 years (18–99 years), and median NIHSS was 4. Sixty-nine patients (32.7%) out of the 211 patients under the age of 60 were diagnosed as mimic. Mimics were significantly more frequent among the young (OR = 3.3, 32.7% vs. 12.8%, p = < 0.001). The most frequent mimics diagnoses among patients under 60 years of age were migraine (11.8%), no definite diagnosis (11.4%) and peripheral vertigo (3.3%). Mimics were independently associated with age < 50 years (OR = 4.97, p = < 0.001), not currently working/studying (OR = 3.38, p = 0.002) and not having aphasia on admission (OR = 2.95, p = 0.025). None of the mimics under the age of 60 years had symptomatic or asymptomatic intracerebral hemorrhage as a complication to thrombolysis.

**Conclusion:**

We found significantly more mimics in the young, of which migraine was the most predominant diagnosis. Thrombolysis with alteplase or tenecteplase did not cause ICH in any mimics under 60 years.

## Introduction

Several studies have shown that stroke mimics occur more often among young patients [1]. Both the incidence of stroke and stroke mimics is expected to increase in young patients [2, 3]. It is therefore important to assess conditions that present mimics, predictors of mimics, and safety of thrombolysis in this group. Few studies have compared the clinical and epidemiological differences between mimics in younger and older patients.

Precise diagnosis of mimics in the acute setting is difficult. Reduced consciousness, lower National Institutes of Health Stroke Scale (NIHSS) [4] score on admission, female sex, lower age, history of migraine, and lower blood pressure are associated with mimics in some previous studies. Mimics also have less vascular risk factors like hypertension, dyslipidemia, ischemic heart disease, atrial fibrillation, peripheral vascular disease, smoking, and diabetes [1]. Earlier publications have designed clinical prediction tools like TeleStroke Mimic Score (TSMS) and Khan Score with the purpose of discerning mimics from stroke [5–7]. The usefulness of such tools is uncertain.

Increasing incidence of stroke among the young can lead to a growing number of young mimics treated with thrombolysis in years to come [2]. Shorter “door-to-needle”-time has been associated with a higher rate of mimics treated with thrombolysis [8]. The proportion of mimics treated with thrombolysis is 2–15% [9–13]. Thrombolysis of mimics with alteplase is generally considered safe, with an intracerebral hemorrhage (ICH) rate of 0-1.2% [9–13]. There has been an increasing interest in the use of tenecteplase in acute stroke [14]. The safety of tenecteplase in mimics is yet to be confirmed.

We present a sub-study of the Norwegian Tenecteplase Stroke Trial (NOR-TEST) [15]. The trial had a high proportion of mimics due to liberal inclusion criteria reflecting the practice of fast approach towards patients with minor stroke symptoms. Our aims were to identify the common mimics in young patients under the age of 60 years who received thrombolysis, to analyze the risk of hemorrhage after treatment with thrombolysis, and to identify risk factors and clinical parameters that might identify mimics in this group.

## Methods

This study is based on data collected in NOR-TEST, a multicenter, randomized open-label, blinded endpoint, phase-3 trial designed to investigate safety and efficacy of tenecteplase vs. alteplase in patients with acute ischemic stroke. Patients were included between 1 and 2012 and 30 September 2016 in 13 stroke units in Norway. Patients with clinically suspected acute ischemic stroke, aged over 18 years, living independently pre-stroke, admitted within 4.5 h of stroke onset with measurable deficits according to NIHSS and judged eligible for intravenous thrombolysis according to European guidelines were included in the trial. Exclusion criteria reflected current evidence regarding contraindications to intravenous thrombolysis. Indication of treatment eligibility and study inclusion was assessed by emergency room clinicians after cerebral non-contrast CT and CT angiography. Patients were randomized to either 0.4 mg/kg tenecteplase or 0.9 mg/kg alteplase. Treatment allocation was blinded to treating physicians and nurses in the stroke unit. Patients received state-of-the-art multidisciplinary stroke unit care according to the Departments’ Standard Operating Procedures [15].

Risk factors and baseline variables including prior ischemic stroke, transient ischemic attack (TIA), myocardial infarction, hypertension, smoking, atrial fibrillation, diabetes mellitus, hyperlipidemia, and working/studying status were registered. Patients diagnosed with either acute cerebral ischemia or TIA were categorized as stroke group, and patients with any diagnosis other than ischemic stroke or TIA as mimics group. Final diagnosis was set by local clinicians based on clinical history, clinical findings, and radiological findings. Patients were grouped post-hoc into young (< 60) and old (≥ 60). Neurological deficits on admission were measured with NIHSS. Cerebral MRI or CT were done routinely 24–48 h after treatment. Symptomatic ICH (sICH) was defined according to the ECASS III criteria as any intracranial hemorrhage (ICH) causally related to an increase of four points or more in the NIHSS score. Radiological evaluation was done by investigators blinded for treatment allocation.

The study was done in accordance with the guidelines for Good clinical practice and the declaration of Helsinki and was monitored by external controllers from Western Norway Health Trust Research. The trial was reviewed and approved by the Regional Committee for Medical and Health Research Ethics and the Norwegian Medicines Agency.

This is a sub-study and for full details regarding methodology see original trial (NOR-TEST) [15].

### Statistics

Mimics group was compared with stroke group with univariate analyses of demographic data and clinical characteristics. Chi-squared test was used for categorical variables. Student’s t test or Mann-Whitney U test were used for continuous variables when appropriate. All parameters from the univariate that turned out statistically significant (p < 0.05) were used in a multivariant analyses with logistic regression. Clinical characteristics were added one by one with stepwise forward method. The initial variables added to the logistic regression were age, sex, systolic and diastolic blood pressure, working/studying status, aphasia, dysarthria, sensory affection, and facial paresis. Variables were added one at the time until no significant increase in the model’s accuracy could be obtained. Sex was not added by the forward method initially but added manually later in an additional model. Statistical analyses were performed using SPPS 28.0.1.0.

## Results

Out of 1107 patients who were originally included in the trial, 7 patients were excluded due to withdrawal of consent or eligibility for thrombolytic treatment reconsidered before randomization. Nine patients originally diagnosed as mimics, were removed because the final diagnose was changed to a cerebrovascular disease after further investigation. Of these patients two patients had carotid artery syndrome, one patient was diagnosed with amaurosis fugax, two patients had retinal occlusion, one patient had unspecified cerebral infarction, two patients had unspecified TIA, and one patient had other specified cerebrovascular disease. None of these patients had ICH.

Out of the 1091 patients included in the trial, 434 (39.8%) were female, median age 77 years (18–99 years), and median NIHSS 4. A total of 181 patients (16.6%) were diagnosed as mimic, 830 (76.1%) as stroke, and 80 (7.3%) as TIA. Of the 1091 patients included in the trial, 211 patients (19.3%) were under the age of 60 years. Sixty-nine patients (32.7%) out of the 211 patients under the age of 60 were diagnosed as mimic. Mimics were significantly more frequent among the young with an odds ratio of 3.3 (32.7% vs. 12.8%, CI = 2.3–4.7, p = < 0.001). The different mimics diagnoses are listed in Table 1. The most frequent mimics diagnoses among patients under 60 years of age were migraine (n = 25, 11.8%), no definite diagnosis (n = 24, 11.4%) and peripheral vertigo (n = 7, 3.3%). Diagnoses that were significantly more frequent among young patients were migraine (11.8% vs. 1.7%, OR = 7.7, CI = 4.0–15.0, p = < 0.001), no definite diagnosis (11.4% vs. 4.4%, OR = 2.7, CI = 1.6–4.7, p = < 0.001), functional neurological disorder (1.9% vs. 0.2%, OR = 8.5, CI = 1.5–46.5, p = 0.003) and demyelinating disease (0.9% vs. 0%, p = 0.004).

Clinical characteristics and risk factors for stroke and mimics under 60 years are shown in Table 2. Mimics were younger, more frequently female, and less frequently working or studying compared to patients with stroke. On admission, mimics had lower systolic and diastolic blood pressure and were less likely to present with aphasia, dysarthria, and facial paresis, whereas loss of sensation was a more frequent symptom among mimics.

**Table 1:** Frequency of different mimic-diagnoses in patients over and under 60 years of age

		Under 60 years of age N (%)	Over 60 years of age N (%)	OR (95 CI)	P-value
**Total**		211 (100)	878 (100)		
**Diagnose**	Migraine	25 (11.8)	15 (1.7)	7.7 (4.0–15.0)	< 0.001
	No definite diagnosis	24 (11.4)	39 (4.4)	2.7 (1.6–4.7)	< 0.001
	Functional neurological disorder	4 (1.9)	2 (0.2)	8.5 (1.5–46.5)	0.003
	Demyelination diseases	2 (0.9)	0		0.004
	Orthopedic	0	4 (0.5)		0.326
	Peripheral nerve disorder	3 (1.4)	6 (0.7)	2.1 (0.5–8.5)	0.287
	Epilepsy	0	8 (0.9)		0.164
	Dementia or delirium	0	1 (0.1)		0.624
	Neoplasm	0	3 (0.3)		0.395
	Toxic metabolic	2 (0.9)	4 (0.5)	2.1 (0.4–11.5)	0.386
	Infection	2 (0.9)	4 (0.5)	2.1 (0.4–11.5)	0.386
	Peripheral vertigo	7 (3.3)	26 (3.0)	1.1 (0.5–2.6)	0.786
	Total Number of cases (% of whole population in age group)	69 (32.7)	112 (12.8)	3.3 (2.3–4.7)	< 0.001

**Table 2:** Univariate analyses of clinical characteristics for mimics and stroke in patients under 60 years of age

	Mimics	Stroke	P-value
Age, mean (SD)	41.8 (10.6)	50.8 (8.3)	< 0.001
Sex, female N (%)	42 (60.9)	49 (34.5)	< 0.001
Risk factors N (%)			
- Currently working/ studying	39 (59.1)	103 (78.0)	0.005
- Hypertension	13 (18.8)	38 (26.8)	0.207
- Diabetes mellitus	3 (4.3)	13 (9.2)	0.216
- Atrial fibrillation	1 (1.4)	4 (2.8)	0.540
- Smoking, current*	24 (40.7)	54 (41.5)	0.911
- Hyperlipidemia	4 (5.8)	15 (10.6)	0.257
- Prior stroke/TIA	5 (7.2)	13 (9.2)	0.642
- Prior heart disease	2 (2.9)	9 (6.3)	0.292
Parameters on admission			
- Systolic blood pressure mmHg, mean (SD)	136.0 (18.6)	147.4 (21.0)	< 0.001
- Diastolic blood pressure mmHg, mean (SD)	82.4 (13.6)	86.6 (13.3)	0.044
- Aphasia N (%)	8 (11.8)	36 (25.5)	0.022
- Sensory affection N (%)	44 (64.7)	67 (47.5)	0.020
- Dysarthria N (%)	16 (23.5)	53 (37.6)	0.043
- Facial paresis N (%)	24 (35.3)	71 (50.4)	0.040
- Gaze palsy N (%)	2 (2.9)	15 (10.6)	0.056
- NIHSS, median (interquartile range)	4 (4)	4 (5)	0.957
- Consciousness N (%)	6 (8.8)	6 (4.3)	0.184
- Orientation N (%)	7 (10.3)	23 (16.3)	0.245
- Commands N (%)	1 (1.5)	8 (5.7)	0.161
- Visual field loss N (%)	10 (14.7)	18 (12.8)	0.700
- Limb motor deficit N (%)	45 (66.2)	90 (63.8)	0.740
- Limb ataxia N (%)	27 (39.7)	43 (30.5)	0.186
- Neglect N (%)	8 (11.8)	18 (12.8)	0.837
- Glucose (mmol/L), mean (SD)	5.90 (1.24)	6.56 (2.41)	0.113
Complications N (%)			
- ICH	0 (0)	3 (2.1)	0.224
- sICH	0 (0)	2 (1.4)	0.322
Imaging N (%)			
- CTA performed on admission	60 (87.0)	138 (97.2)	0.004
- MRI performed	68 (98.6)	130 (91.5)	0.047

Abbreviations: SD, standard deviation; TIA, Transient ischemic attack; NIHSS, National Institutes of Health Stroke Scale; sICH, symptomatic intracerebral hemorrhage; CTA, computed tomography angiography; MRI, magnetic resonance imaging

Logistic regression analysis with mimics vs. stroke as dependent variable is shown in Table 3. Mimics were independently associated with age < 50 years (OR = 4.97, 95% CI = 2.32–10.65, p = < 0.001), not currently working/studying (OR = 3.38, 95% CI = 1.56–7.33, p = 0.002) and not having dysphasia on admission (OR = 2.95, 95% CI = 1.14–7.61, p = 0.025). Other non-significant variables associated with mimics were lower systolic blood pressure (OR = 0.92 per 5 increase in mmHg, 95% CI = 0.84–1.10, p = 0.078) and female sex (OR = 1.95, 95% CI = 0.95-4.00, p = 0.067).

None of the mimics under 60 years of age had symptomatic or asymptomatic ICH as a complication to thrombolysis. In patients with stroke, any ICH occurred in 3 patients (2.1%), of whom 2 (1.4%) had symptomatic ICH (sICH) and 1 (0.7%) asymptomatic ICH (aICH). One patient with sICH had NIHSS 2 on admission, NIHSS 12 after two hours, and CT control showed hemorrhagic transformation within the ischemic area (PH2). A second patient with sICH had NIHSS 2 on admission, NIHSS 5 after two hours, and CT after three hours showed parenchymal hemorrhage remote from the infarcted area (PHr). One patient with aICH had NIHSS 15 on admission, CTA showed a left M1 occlusion, and MRI after 24 h showed ischemic infarction with hemorrhagic transformation within the ischemic area (PH1).

**Table 3:** Logistic regression analysis with mimics vs. stroke as dependent variable in patients under 60 years of age

	Odds ratio	95% CI	P-value
Age < 50 years	4.97	2.32–10.65	< 0,001
Systolic BP on admission	0.92 (per 5 increase in mmHg)	0.84–1.10	0.078
Not currently working/studying	3.38	1.56–7.33	0.002
No aphasia on admission	2.95	1.14–7.61	0.025
Female sex	1.95	0.95-4.00	0.067

Pseudo R-square: 0.320

## Discussion

Our study showed a higher proportion of mimics among young patients. Migraine, functional neurological disorders, demyelinating diseases and “no definite diagnosis” were more common among patients under the age of 60 years compared to older patients. Clinical and demographic variables associated with mimics in young patients were sensory symptoms, lower blood pressure, female gender, currently not working/studying, and absence of dysarthria, facial palsy and aphasia. The variables that were independently associated with mimics were absence of aphasia, female gender, and working/studying status. We also found no difference in NIHSS score between mimics and stroke in young patients.

In line with previous studies, we found that migraine was the most common mimic among the young [1]. Tu et al. found young age and migraine to be the strongest predictors of mimics [5]. One must however, be careful when evaluating patients with suspected stroke and history of migraine. Oygarden et al. found a lower rate of thrombolysis to young stroke patients with migraine compared to young stroke patients without migraine [16]. This is most likely a result of misinterpreting stroke as a migrainous phenomenon. Younger patients are more exposed to this misinterpretation due to the relatively lower rate of stroke and higher rate of migraine [2, 17].

Not currently working/studying was independently associated with mimics for patients under 60 years. We have not found any other studies that reports a similar finding. One explanation could be that migraine is one of the most important reasons for work disability among young people, and especially among young women [18]. Although statistically not significant, the proportion of patients not currently working/studying was higher in the migraine group than the rest (41.7% vs. 26.4%, OR = 1.99, 95% CI = 0.83–4.79, p = 0.12). Because of the small sample size, one must be careful drawing conclusions from this finding.

Some previous studies show that less severe stroke symptoms and lower systolic blood pressure are associated with mimics [1, 19, 20]. Our study failed to show such an association, although systolic blood pressure tended towards being significant in the multivariant analysis. This discrepancy may be explained by the low median NIHSS in NOR-TEST and generally lower mean systolic blood pressure and less severe stroke symptoms in young stroke patients [21, 22]. Contrary to previous studies, we did not find a higher frequency of aphasia among mimics [9, 19, 20].

Even though some factors are associated with mimics, there are no reliable symptoms or clinical signs, which can consistently identify mimics with precision. Mimics prediction tools like TSMS and Khan Score make use of a certain variables to predict mimics [5–7]. The variables included in TSMS are age, medical history of atrial fibrillation and seizures, hypertension, presence of facial weakness, and NIHSS above or below 14. TSMS, despite having high sensitivity of 91%, is a poor tool in identifying mimics due to its low specificity of 59% [5]. Kahn score is showed to have a high specificity of 88.5–100% depending on the cut off value, and a positive predictive value (PPV) of 97.5% [5, 6]. In harmony with our findings, Khan score includes history of migraine a predictor for mimics. The other variables included in Khan score are the same as TSMS expect facial weakness and NIHSS score. Given its specificity and PPV, Khan score may be the preferred clinical score before deciding to withhold thrombolysis or not. On the other hand, it has a low sensitivity (32.1%) and therefore a poor ability to rule out mimics [5]. Our study found only three out of the seven factors used in TSMS and Khan Score to be associated with mimics. Mimics prediction tools alone is not sufficient to exclude patients from potentially beneficial thrombolysis. Using advanced imaging like CT perfusion and MRI in the emergency room could be of help in such cases [23].

None of the mimics under the age of 60 years had a symptomatic or asymptomatic ICH as a complication to thrombolysis. This is in line with previous studies, which found a low frequency of adverse complications ranging from 0 to 1.2% among mimics [9–13]. In NOR-TEST, patients were assigned to either 0.4 mg/kg tenecteplase or 0.9 mg/kg alteplase. As standard the standard dose of tenecteplase trends towards 0.25 mg/kg [24, 25], there is reason to believe complications to be minimal in the future. This supports a liberal attitude toward reperfusion treatment to young patients with suspected stroke.

The strength of this study includes the use of data from a randomized, controlled trial with a high number of mimics. One weakness is that this is a retrospective analysis of prospectively included patients. The sample size was relatively small and therefore the study has a limited statistical power. Some patients were also removed because of withdrawal of consent, and therefore there is some consent bias. The absence of vascular risk factors in patients diagnosed with mimics and presence of ICH after thrombolysis in patients diagnosed as stroke are possible biases in the diagnostic evaluation. Finally, 8.5% of patients under 60 years diagnosed with stroke did not have a follow-up MRI. The real number of mimics may therefore be considered an approximation. The lack of a standardized diagnostic process of stroke mimics may be a weakness but is inherent in any study of mimics. The correct diagnosis is of special importance when assessing ICH in stroke mimics. We therefore reviewed all patients with ICH to provide evidence that bleeding complications were related to patients with an ischemic event.

In conclusion, we found significantly more mimics in the young, of which migraine was the most predominant diagnosis. The variables independently associated with mimics were younger age, absence of aphasia, and currently not working/studying. It is difficult to identify mimics reliably using risk factors and clinical parameters alone. None of the mimics had an ICH after treatment with alteplase or tenecteplase. In a wider research context, we conclude that there is no compelling reason to withhold thrombolysis out of fear of erroneously treating young mimics after non-contrast CT imaging in the emergency department.

## Data Availability

All data used in the article is gathered from NOR-TEST [15]. The datasets used and/or analysed during the current study are available from the corresponding author on reasonable request.

## Abbreviations

NIHSS: National Institutes of Health Stroke Scale
TSMS: TeleStroke Mimic Score
ICH: Intracerebral hemorrhage,NOR-TEST,Norwegian Tenecteplase Stroke Trial
TIA: Transient ischemic attack
ECASS: European Cooperative Acute Stroke Study
OR: Odds ratio
CI: Confidence interval,SD,standard deviation
PPV: Positive predictive value
